# Methanol Extract of *Coleus amboinicus* (Lour) Exhibited Antiproliferative Activity and Induced Programmed Cell Death in Colon Cancer Cell WiDr

**DOI:** 10.1155/2020/9068326

**Published:** 2020-01-24

**Authors:** Farida Laila, Dedi Fardiaz, Nancy Dewi Yuliana, M. Rizal M. Damanik, Fitriya Nur Annisa Dewi

**Affiliations:** ^1^Department of Food Science and Technology, Bogor Agricultural University, IPB Dramaga Campus, Bogor 16680, Indonesia; ^2^College of Vocational Studies, Bogor Agricultural University (IPB University), Jalan Kumbang No. 14, Bogor 16151, Indonesia; ^3^Department of Community Nutrition, Faculty of Human Ecology, Bogor Agricultural University, IPB Dramaga Campus, Bogor 16680, Indonesia; ^4^Primate Research Center, Bogor Agricultural University, Jalan Lodaya II/5, Bogor 16151, Indonesia

## Abstract

*Coleus amboinicus*(Lour) (CA) has been reported to possess many pharmacological activities. In this study, evaluation of cytotoxicity using brine shrimp lethality bioassay and MTT assay using WiDr cell lines was carried out. The expression of several genes responsible for programmed cell death of the methanol extract of CA was also investigated. The morphology of the cells undergoing apoptosis was detected using Hoechst staining assay. The gene expression of BAX, *BCL2*, *P53*, *Caspase 1, 7, 8,* and *9* of treated samples with different concentrations (10, 15, 25 & 50 *µ*g/ml) were measured with RT PCR. The phytochemical profiles were investigated using LC MS. The results showed that the lethality concentration (LC_50_) of methanol extract using brine shrimp was 34.545 *µ*g/ml and the extract exhibited good antiproliferative activity against cancer cells WiDr with IC_50_ value (8.598 ± 2.68 *µ*g/ml) as compared to standard drug 5-fluorouracil (IC_50_ value 1.839 ± 0.03 *µ*g/ml). There was apoptotic evidences from the morphology of treated cells. The expressions of *BAX*,*P53*, and *Caspase *9 were upregulated in lower concentration of the extract (10 and 15 *µ*g/ml) but downregulated in higher concentration (25 and 50 *µ*g/ml). *BCL2* as anti-apoptotic gene was downregulated in all concentrations. *Caspase 1* and *Caspase 7* were upregulated in high concentration (25 and 50 *µ*g/ml), but downregulated in lower concentrations. These data provide a mode of cell death for the methanol extract of CA in low concentrations corresponding to apoptosis with intrinsic pathway. Many valuable compounds identified including caffeic acid, rosmarinic acid, malic acid, eicosapentanoic acid, benserazide, alpha-linolenic acid, betaine, Salvanolic B, 4-hydroxibenzoic acid and firulic acid have been previously reported as being active agents against many cancer cells. This study suggested that CA might become an effective ingredient for health-beneficial foods to prevent colon cancer.

## 1. Introduction

Colorectal cancer is the third most common cancer in men and women and the fourth cause of death from cancer. Lifestyle factors like consuming red meat, processed meat, alcohol drinks, being overweight, or obese affect the risk of developing colorectal cancer [1].

The accumulation of genetic errors and some of which affect the control of apoptosis is the result of the increase in colorectal cancer [2]. Apoptosis is a programmed and regulated cellular process and triggered by physiological and pathological conditions [3]. The development of cancer is deeply affected by apoptosis process and its related pathway. Therefore, triggering apoptosis is one of the goals of cancer prevention [4]. The anticancer properties which have been revealed in many studies were the phytochemical content in plant responsible for apoptotic activity and have the potential to prevent, reverse and/or inhibit progression of cancer [5].

*Coleus amboinicus* (Lour) (synonym: *Plectranthus amboinicus*) belongs to the family of Lamiaceae is fleshy and highly aromatic. This plant has a common name as Mexican mint, Spanish thyme, Cuban oregano, or Indian borage. The leaves are used to add flavor to meat and bean dishes, especially in Carribean cuisine [6]. In Indonesia, this plant is called torbangun and has been used traditionally for hundreds of years by Bataknese people as a breast milk stimulant (a lactagogue) [7]. The pharmacological activities of *Coleus amboinicus* (Lour) have been widely studied. A study has reported that antihyperglycemic and anti-hyperlipidemic activity of torbangun in Streptozotocin induced diabetic rats [8]. Significant anticonvulsant activity of *C. amboinicus* in Swiss Albino Mice was also reported and the presence of flavonoids, alkaloids, and saponin in the extract may be responsible for this activity [9]. *Plectranthus amboinicus* Lour also had a cytotoxic effect on HeLa cells [10]. However, the activity and the mechanism of action against colon cancer cell in different concentrations of extract have not been performed.

The present study was designed to investigate the cytotoxicity and anticancer activity of methanol extract of *Coleus amboinicus* (CA) and also to describe the molecular mode of action in many concentrations of treated cells from the expression of several genes that were responsible for programmed cell death. The phytochemical contents were also investigated to evaluate the presence of important compounds that contributed to anticancer activity.

## 2. Material and Methods

### 2.1. Chemicals

Roswell Park Memorial Institute 1640 (RPMI-1640), Phosphate Buffer Saline (PBS), 3-(4,5-dimethylthiazol-2-yl)-2,5-diphenyltetrazolium bromide (MTT), were purchased from Sigma Aldrich chemical Company (St. Louis, MO, USA). 100,000 mg/L streptomycin and 100x antibiotic solution (100,000 U/I of penicillin), trypsin, acetone, hexane, 5-fluorouracil, formaldehyde, potassium chromate, and DMSO were purchased from The Merck Company (Germany). *Sea sand *(*methanol washed* 20–35 mesh) was purchased from Waco (Japan). Primer gene caspase 3/7, 8, 9, Bax, Bcl-2, *housekeeping* gene *β*-actin and p53 were purchased from Integrated DNA Technologies (Singapore). *RNEeasy minikit* was purchased from Qiagen (Singapore). *Superscript III RT* was purchased from Invitrogen (Singapore), *SsoFast Evagreen Mastermix* was purchased from Bio-Rad Laboratories (Singapore). Nuclease free water DEPC-treated PCR grade and Hoechst 33342 were purchased from Thermo Fisher Scientific (Singapore).

### 2.2. Collection and Extraction the Plant Material

*Coleus amboinicus* leaves were collected from Bogor Agricultural University Teaching Farm. The samples were freeze dried for 48 hours and grinded using *Knife Mill Grindomix GM 200* at 10 × 1000 rpm for 20 s. In brief, Accelerated solvent Extractor system (Dionex ASE 350) was used to extract the samples using methanol. For solvent evaporation, a centrifugal evaporator (CVE-200D EYELA) was employed.

### 2.3. Brine Shrimp Cytotoxicity Study

The cytotoxicity of CA was conducted using Brine Shrimp Lethality Assay (BSLA) by the method of Meyer et al. [11] with several modifications. Brine shrimp eggs (*Artemia Salina* Leach) were permitted to hatch as larvae (*nauplii*) in artificial sea water under light and good aeration. After 48 h, the larvae were transferred to plate-24 well plates and added with 1.0 ml sea water. The solution of extract was added until the concentration extract in wells of 1000, 500, 250, 125, 50, and 10 *µ*g/ml. Negative control was made by using 2 ml artificial seawater without adding the extract. Potassium chromate K_2_CrO_4_ (Sigma Aldrich, St. Louis, MO, USA) was used as positive control with concentrations of 5.0, 10.0, 15.0, 20.0, and 25.0 *µ*g/ml in artificial sea water. The numbers of surviving *larvae* in each well were counted after 24 h. The concentration that killed 50% of the *nauplii* (LC_50_) and 95% confidence interval were calculated by Graphad using Probit analysis. The plant samples were weight three times and the procedure was performed in triplicate for each plant sample.

### 2.4. Cell Culture

WiDr cell lines were obtained from Primate Center Research, Bogor Agricultural University and cultured in Roswell Park Memorial Institute 1640 (RPMI-1640) (Sigma Aldrich, St. Louis, MO, USA) and supplemented with (5% PBS penicillin and 1% streptomycin (Merck Company Germany) at 37.0°C and 5% CO_2_. Based on the chromosomes and isozymic evidence, the cell line WiDr (ATCC® CCL-218™) is a derivative of another colon cancer cell line, HT-29 (ATCC® HTB-38™) [12].

### 2.5. Cancer Cell Viability Assay

The antiproliferative activity of methanol extract of CA was studied using MTT assay. Cells that have undergone confluence were cultured, and the media were removed and the flask was cleaned from the media using 10 ml PBS and after that PBS was removed from the flask. 5 ml of Trypsin (0.125%) was added to the flask and incubated at 37°C for 5 min. The released cells from the substrate were inserted into a 15 ml tube and centrifuged 1500 RPM for 5 min. Supernatants were removed and cells were counted using the haemacytometer.

The cells were seeded in the density 2 × 10^4^ cells/well and incubated overnight at 37°C and exposed to methanol extract of CA. The cells were treated with various concentrations (1–100 *µ*g/ml in 0.1% DMSO) to find the concentration of extract that inhibits 50% of the cell growth (IC_50_ value). After incubation for 48 h, the cells were washed with PBS to remove any traces of samples, then added with 10 *µ*l of 5 *µ*g/ml MTT and incubated for 4 h at 37°C and 5% CO_2_. Finally, the medium was removed and 100 *µ*l of 95% ethanol was added to dissolve the formazan crystals. The plate was analyzed by using a microplate reader at 595 nm (Thermo Fisher, Waltham, MA, USA). The inhibition of the samples to WiDr cell lines was calculated using formula:

(1) $$\begin{array}{c} \%\mathrm{inhibition}=\frac{\text{ODsample}-\text{ODcontrol}}{\text{ODcontrol}}\times 100. \end{array}$$

### 2.6. Detection of Apoptosis Using Hoechst Staining Method

WiDr cells were seeded at a density 5000 cells/well into the Chamber slide system 8 well. The cells were treated with methanol extract and 5-fluorouracil at 5 and 15 *µ*g/ml and negative control (without sample) and incubated at 37°C and 5% CO_2 _for 48 h.The cells were washed with 500 *µ*l PBS twice followed by fixation with 10% formaldehyde and incubated for 1 h and then washed again with PBS. The cells were stained with 100 *µ*l Hoechst 33258 which has been diluted 1000×, and then incubated for 30 min at room temperature, and washed again with PBS. The chromatin structure of the cells was observed by fluorescence microscopy (Nikon Optivat-2) with 365/460 nm excitation/emission that connected with Digital Imaging System (Dino eye Software, Anmo Taiwan).

### 2.7. Gene Expressions

The expressions of genes that related to apoptosis were measured by Real time PCR. The WiDr cells were seeded in 12-well plates with RPMI 1640 and 10% PBS and incubated at 37°C and 5% CO_2_ for 24 h. After that, the cells were treated with methanol extract at 10, 15, 25, and 50 *µ*g/ml concentration, untreated cells as a negative control and 5-fluorouracil in IC_50_ concentration as positive control, and then incubated for 48 h. The extraction of RNA from treated and untreated cells was done using Commercial RNeasy mini kit (Qiagen, Germany). The quantity of RNA was evaluated by Nanodrop 2000C UV Spectrophotometer. The total RNA was reversed to cDNA by using Superscript III First Strand Synthesis System for RT-PCR. The gene expression levels were normalized with *β*-actin reference gene. The specific primers including BAX, BCL2, P53, *Caspase* 1, 7, 8, and 9 used in the real time PCR technique was carried out using *SSo fast evagreen Supermix* according to the manufacturer's protocols. Primer sequences and annealing temperature for quantitative PCR are shown in Table 1. Melting curves were checked to validate the PCR specificity. The relative expression of each gene was calculated using the 2^−∆∆Ct^ method.

### 2.8. Phytochemical Profiles

The chemical composition of methanol extract of CA was determined using LC UHPLC Vanquish Tandem Q Exactive Plus Orbitrap HRMS (ThermoScientific). The sample was separated on a column of Accucore phenyl hexyl, 100 × 2.1 mm, 2.6 *µ*m. The flow rate was set at 0.3 ml/min, using H_2_O + 0.1% formic acid (A) and acetonitrile (B) as an eluent. The gradient was set at 0–1.5 min of 5% B, 1.5–9 min of (5–9% B), 9–13 min of (10–20% B), 13–17 min of (20–28% B), 17–23 min (28–70% B), 23–26 min (70–95% B), 26–29 (95% B), 29–32 (5% B). The detections of compounds were performed with mass range at 80–2000 m/z. The chemical compounds were identified using ThermoScientific™ Compound Discoverer™ software.

### 2.9. Statistical Analysis

The LC_50_ value of BSLA test using Probit analysis and IC_50_ of antiproliferative cells using MTT assay were determined by Graphad 8.01 (GraphPad Software, San Diego, CA, USA). The Gene expression results were determined using One Way ANOVA with Dunnet's test to evaluate the significance of differences in fold changes between cell with treatment and control. The statistical significance was set at *p* < 0.05.

## 3. Results and Discussion

### 3.1. Cytotoxicity Using Brine Shrimp Lethality Assay (BSLA)

**Figure 1:**
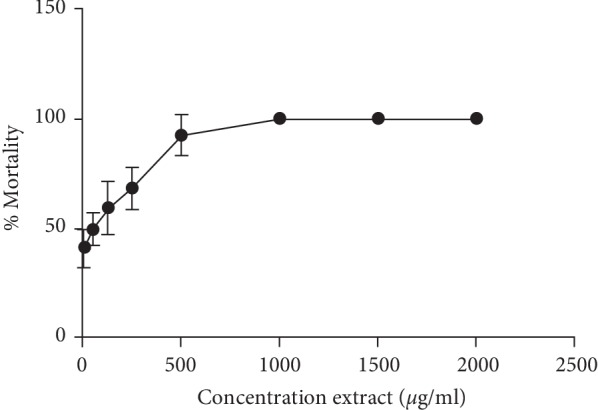
Mortality percentage (%) curve of brine shrimp exposed to different concentrations of methanolic extract of Coleus amboinicus.

The preliminary step of this study was to determine the cytotoxicity of methanol extract of CA using BSLA. The result showed, the methanol extract of CA caused an increase in % mortality of shrimp in a dose dependent manner (Figure 1). One hundred percent mortality was observed at 1000 *µ*g/ml and above. The result of lethality concentration (LC_50_) of extract using Probit test was 34.545 *µ*g/ml with 95% confidence interval of 0.731 to 1.432. This result was higher than positive control potassium chromate (LC_50_ 5.520 *µ*g/ml). A previous study suggested that LC_50_ of the extract or pure compounds in the brine shrimp test less than 100 *µ*g/ml is categorized as a potential cytotoxic and toxic substance [13]. Based on the result, the LC_50_ value of methanol extract of CA was fallen to its criteria and indicated the presence of potent cytotoxic compounds and probably antitumor agents in the methanol extract of CA.

Brine Shrimp Lethality Assay has been suggested as a valid method of evaluation of cytotoxicity and it can be extrapolated for cell lines toxicity and tumor activity [14]. A previous study has used BSLA for preliminary screening to find the cytotoxic activity of *Markhamia tomentosa* before cytotoxicity test using cell lines [15]. Another study also investigated the cytotoxicity of the rhizome of several medicinal plants using BSLA to search which rhizome can be used as a source of cytotoxic agent [16]. The cytotoxic activity of different extracts of *cyanthea* species using several solvent was also evaluated using BSLA and it was found that ethanol extract was more effective against brine shrimps and the result was subjected to proliferation assay in the cancer cell [17]. BSLA is only for preliminary assessment of toxicity and have been suggested for screening pharmacological activities of plant extract [18]. Therefore, for more precise evaluation of cytotoxic effect, an in vitro anticancer activity test using cell lines should be used.

### 3.2. Antiproliferative Activity

The further study was conducted to determine the inhibition of cell proliferative activity against WiDr cells of methanol extract of CA and standard cytotoxic drug 5-fluorouracil as positive control in various concentrations (0–100 *µ*g/ml) using viability assay. The result of IC_50 _value of the extract from the curve of % inhibition versus concentration was 8.598 ± 2.68 *µ*g/ml [19]. These findings suggested that the methanol extract of CA exhibited anti-proliferative activity in WiDr cancer cells and has a potential as chemotherapeutic agents. A previous study has demonstrated anti-proliferative effect of *P. amboinicus* leaves using MTT assay on MCF-7 human mammary cancer cells with the most active fraction was from chloroform fraction (IC_50_ value of 2.46 *µ*g/ml) and the hexane fraction was 8.85 *µ*g/ml) [10].

The control positive of the MTT assay study was using 5-fluorouracil (5-FU). We found the IC_50_ value of 5-FU was 1.839 ± 0.03 *µ*g/ml. From this result, we suggest that the inhibition of cell proliferative activity of 5-FU was higher than the extract of CA. 5-FU is a common drug used for the treatment of various cancers, including colorectal and breast cancer [20]. 5-FU can inhibit the progression of cancer cells by targeting thymidylate synthase enzyme and promote cell cycle arrest and apoptosis [21]. The side effect of 5-FU to its cytotoxicity were serious and only 10–15% of patients with colorectal cancer could positively respond to 5-FU treatment [21]. There were many studies which combine natural compounds with 5-FU to enhance anti-proliferative activity and reduce 5-FU doses. Previous study reported the combination of 5-FU with Asian ginseng berry polysaccharide could reduce 5-FU doses and increase the anti-proliferative activity of colorectal cancer significantly [22].

### 3.3. Detection of Apoptosis

**Figure 2:**
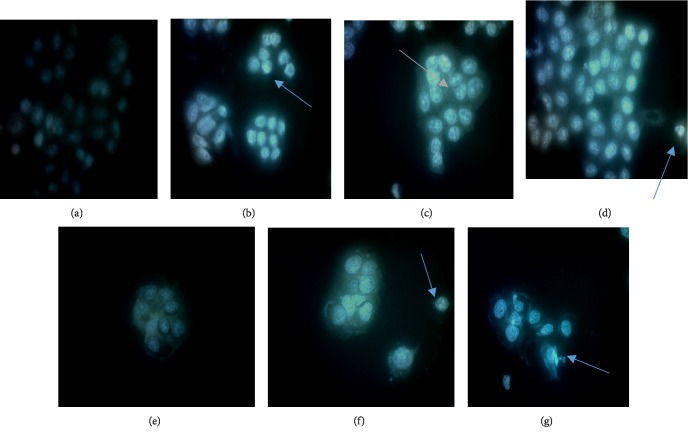
Morphological changes observed by Hoechst staining. (a) Untreated cells showed low fluorescent and normal structures. (b–d) are cells treated with 5-fluorouracil at 5, 15, and 50 *µ*g/mL concentrations respectively. (e–g) are cells treated with methanol extract of CA at 5, 15 and 50 *µ*g/ml concentrations respectively.

A further study was conducted to confirm the pro-apoptotic effect of methanol extracts of *C. amboinicus*. Identification of specific morphological structures was used in order to distinguish apoptotic cells from normal cells. Cell rounding and shrinkage, nuclear fragmentation, membrane blebbing, condensation of chromatin, and apoptotic body formation were identified in apoptotic cells [23]. Florescent images of WiDr cells were treated with Hoechst 33342 after 24-h incubation with 5-fluorouracil and the methanol extracts of CA at different concentrations are shown in Figure 2. The WiDr cells treated with all concentrations of methanol extract of CA showed typical characteristics of apoptosis with clear blue light colony implying DNA fragmentation in the nucleus which indicates apoptosis, and it was seen that the apoptotic effect enhanced at higher doses. The results were compared with positive control 5-FU, which showed a higher apoptotic effect in all concentrations. Hoechst staining indicated apoptotic cell to have shrunken, condensed, and also fragmented after exposure of the extract and 5-F for 24 h. This contrast with the untreated cells showed a low fluorescence, normal nuclei and dispersed chromatin. These results suggest that CA and 5-F induces apoptosis of WiDr cell might contribute to reduce cell viability.

The apoptosis involves several types of mechanisms which include chromatin condensation, the fragmentation of DNA to form vesicles known as “apoptotic bodies”. The reason why apoptotic bodies are not easy to detect and only seen under special condition is because phagocytic cells usually engulf apoptotic cells before apoptotic bodies occur.

### 3.4. Gene Expression

The further study was designed to find the mechanism of apoptosis by evaluating the changes in the expression level of apoptosis-related genes. This study is important for the development of treatment strategies against colon cancer. Changes in expression level of apoptosis–related genes such as *P53*, *BAX*, *BCL2*, *Caspase 1, 7, 8,* and *9* which had been treated with methanol extract of CA at concentrations of 10, 15, 25, and 50 *µ*g/ml were investigated using Real Time PCR to investigate the mechanism of action. Beta actin was served as the housekeeping control in all experiments. We also compared the expression of all related genes to the expression of 5-fluorouracil at IC_50_ concentration. The results of gene expression data a shown in Table 2.

**Figure 3:**
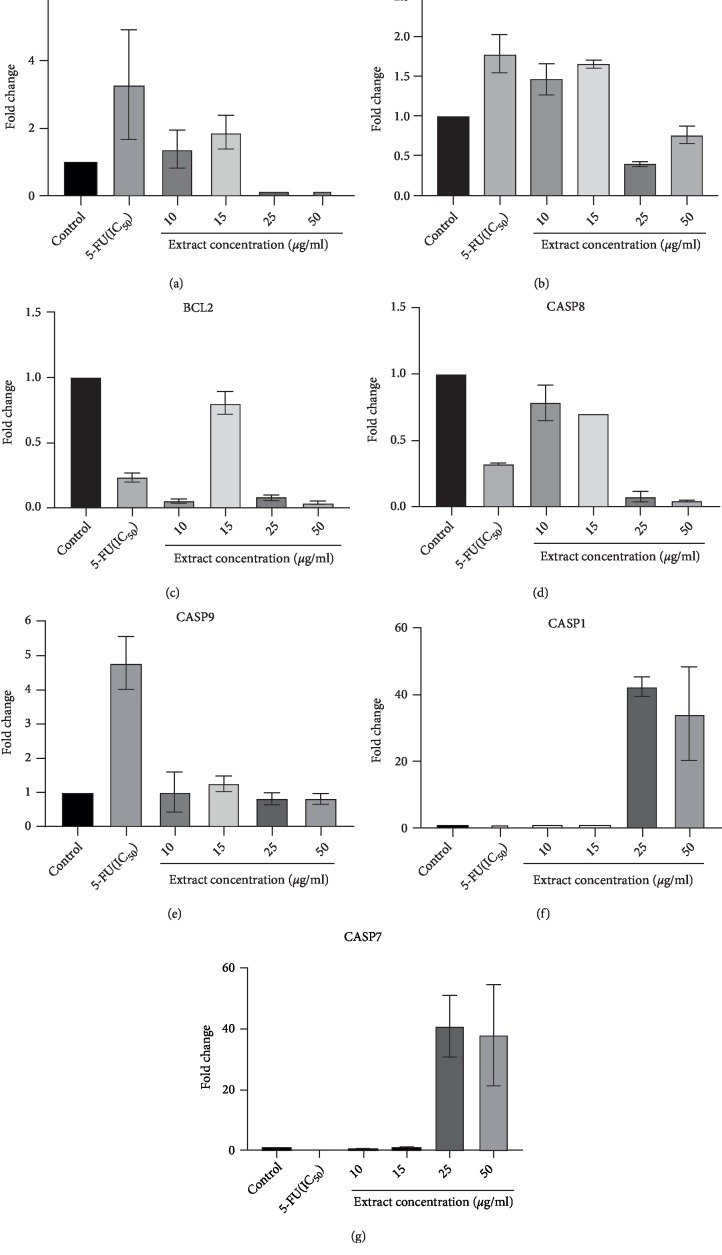
Gene expression of Bax (a); Bcl-2 (b); p53 (c); caspase 8 (d); caspase 9 (e); caspase 1 (f) and (g) caspase 7 of WiDr cells treated with methanol extract at concentrations 10, 15, 25, and 50 *µ*g/ml and compared to the expression of 5-fluorouracil (5-F) at IC50.

Figure 3(a) shows significant upregulation of p53 in WiDr cell treated with 15 *µ*g/ml to control, but downregulated at 25 and 50 *µ*g/ml concentrations. One of the most important molecules in determining oncogenic transformation from the cancer treatment is tumor suppressor protein p53. More than 50% of human cancers related to the defect in this gene [3, 24]. The over-expression of p53 is required for the execution of apoptosis of cancer cells. From this result, we concluded that the mechanism of cells death corresponds to apoptosis only at low concentrations, but it was not happening at higher concentrations.

Figures 3(b) and 3(c) shows there was significant upregulation of *BAX* in WiDr cell treated with 10 and 15 *µ*g/ml of CA methanol extract (*p* < 0.05) compared to control, but at 25 and 50 *µ*g/ml the gene was downregulated. In contrast to *BAX*, the expression *BCL2* was significantly downregulated (*p* < 0.05) for all concentrations of the extract. The expression of *BAX* and *BCL2* of 5-fluorouracil (5-FU) as positive control showed similar results with the extract at lower concentrations (10 and 15 *µ*g/ml).

Two common pathways in apoptosis are mitochondrial or intrinsic pathway and death receptor or extrinsic pathway. Mitochondrial pathway is regulated by Bcl-2 family protein consisting two main groups, namely pro-apoptotic protein and anti-apoptotic protein [3]. Apoptosis process is determined by the balance between pro-apoptotic and anti-apoptotic protein. Mitochondria dysfunction was achieved when the expression of antiapoptotic gene *BCL2* was inhibited and pro-apoptotic gene BAX was up-regulated [25]. From the result, we found the methanol extract of CA initiated apoptosis process at lower concentrations because of the inhibition of BCL2 expression and over-expression of BAX.

The expression of several caspases related to cell death was also investigated in this study. Activation of caspases is the final stage of apoptosis process. Caspases acted as the initiators and executioners and important to the mechanism apoptosis [3].  Figure 3(d) shows the expression of *Caspase 8* was significantly downregulated for all concentrations (*p* < 0.05).  Figure 3(e) shows upregulation of *Caspase 9* in WiDr cell treated with 10 and 15 *µ*g/ml of CA extract compared to control. On the other hand, *Caspase 9* was downregulated at 25 and 50 *µ*g/ml. 5-FU also showed significant upregulation of *Caspase 9* (*p* < 0.05), but *Caspase 8* was downregulated compared to control.

In extrinsic pathway, apoptosis is triggered by extracellular ligan-induced activation of death receptors which leads to activation of *Caspase 8*. Intrinsic or mitochondrial pathway is initiated within the cells and strongly regulated by a Bcl-2 family, which leads to activation of *Caspase 9* [26]. Both pathways culminated the activation of “executioner” caspases, Caspase 3 and Caspase 7 [27]. The results of the expression of *Caspase 8* and *Caspase 9* in the methanol extract to WiDr cell suggested that the mechanism of apoptosis in low concentrations corresponded to intrinsic pathway. This result was also in line with 5-fluorouracil at IC_50_ concentration. The diminished activity of *Caspase 9* in higher concentration is possibly due to the other cascades related to apoptosis or perhaps following the other mechanism of programmed cell death. These results were also in line with the expression of *BAX* and *P53* that upregulated for 10 and 15 *µ*g/ml concentrations and downregulated in 25 and 50 *µ*g/ml concentrations. The caspase activities in WiDr showed that at different concentrations, various caspases were activated.

Interestingly, in 25 and 50 *µ*g/ml concentrations of the extract, Caspase 7 as the executioner was highly expressed. On the other hand, the expression of this gene was downregulated in lower concentration (Figure 3(f)). The result was possible because there might be caspase 3 as another executioner caspase was activated at lower concentrations. *Caspase 8* and 9 can activate both *Caspase 3* and 7 during intrinsic and extrinsic pathways respectively [28].

Many studies have suggested the induction of apoptosis related to colorectal cancer cells based on the expression of several genes. Thunder God Vine extract initiated apoptosis in HTB-39 colon cancer cells, which was linked with upregulated expressions of *BAX* and downregulation of *BCL2* [29]. A sulfated polysaccharide isolated from *C. fulvescens* inhibits the growth of HT-29 colon cancer cells and activated *caspase* 3, 8, and 9 [30]. The roots of *Codonopis bulleynana Forest ex diels* or Tsoong upregulated apoptosis-related genes such as *caspase 3*, *caspase 6*, and *Apaf-1* in HCT116 and SW 480 colon cancer cells [31].

The expression of *Caspase 1* as one of inflammatory caspases was also evaluated in this study. Figure 3(f) shows that *Caspase 1* was downregulated compared to control at 10 and 15 *µ*g/ml. However, at 25 and 50 *µ*g/ml concentrations, *Caspase 1* was significantly upregulated (*p* < 0.05). It also happened to *Caspase 7* which downregulated at 10 and 15 *µ*g/ml concentrations, but upregulated very significantly at 25 and 50 *µ*g/ml. The result was also compared with 5-fluorouracil and it was found that Caspases 1 and 7 were also downregulated at IC_50_ concentration. The activation of Caspase 1 is initiated by the formation of a cytosolic complex called “inflammasome” [32]. Therefore, there is the possibility that at high concentrations of CA methanol extract, the cells were not induced apoptosis but other programmed necrosis. In this study, we found that Caspase 7 was also upregulated significantly in 15 and 50 *µ*g/ml just like Caspase 1. We suggested there might be a connection between Caspase 1 and Caspase 7 in the inflammatory type of cell death. A study reported caspase 7 activation observed is known to induce activation of caspase 1. On the other hand, activation of caspase 3 did not required induction of caspase 1 and the inflammasome [33].

Another study also reported that many anticancer agents at lower concentrations cause apoptosis while at higher doses they cause necrosis [34]. A subclass of another form of cell death, besides apoptosis that are controlled by specific pathways which morphologically discrete from apoptosis called programmed necrosis, has been defined recently [35]. Induction of the programmed necrosis could be useful when drug fails to induce apoptosis. For example, the ability to employ nonapoptotic cell death might provide new opportunities to control cell death and to destroy apoptosis resistant cancer cell [36]. From this study, we could not conclude the mechanism of cell death of the cancer cell lines treated with CA at high concentration and further studies are needed to validate the mechanism of action of other programed cell death.

### 3.5. Phytochemical Profiles of Methanol Extract of C. amboinicus

**Figure 4:**
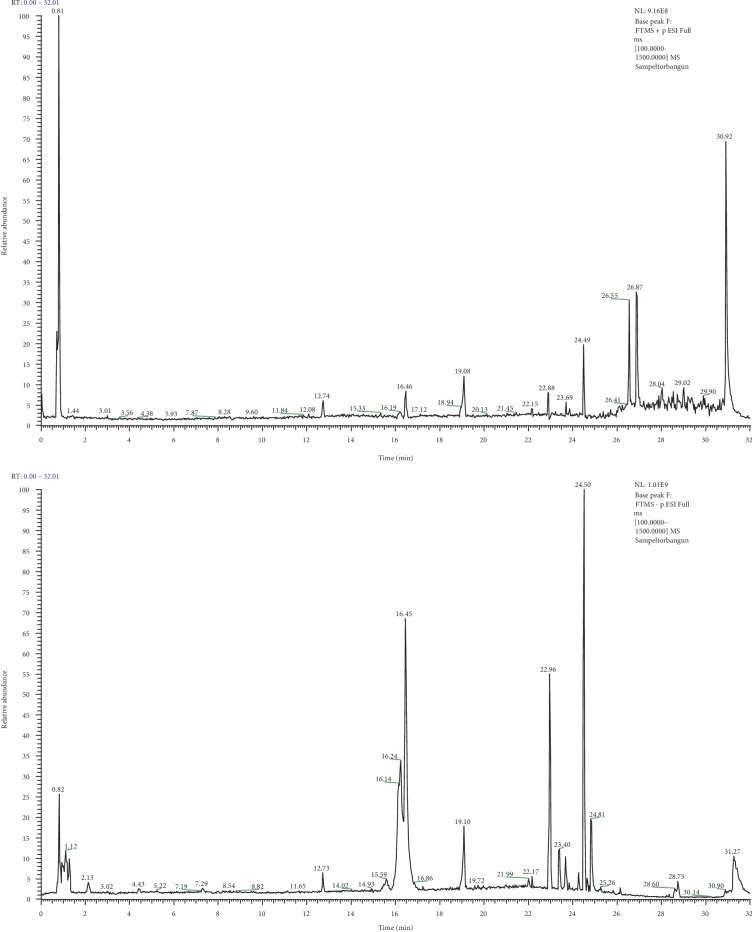
The Chromatogram of methanol extract of CA using Liquid Chromatography–Mass Spectrometry.

The LC MS/MS was used to investigate the chemical profiles of CA. The identified chemicals are shown in Table 3 and the chromatogram is shown in Figure 4. There were many important compounds in the extracts identified including caffeic acid, rosmarinic acid, malic acid, cis-5,8,11,14,17-eicosapentanoic acid (EPA), benserazide, α-linolenic acid, betaine, Salvanolic B, 4-hydroxybenzoic acid, and firulic acid. These compounds have been studied in many literatures for their anticancer activity.

**Figure 5:**
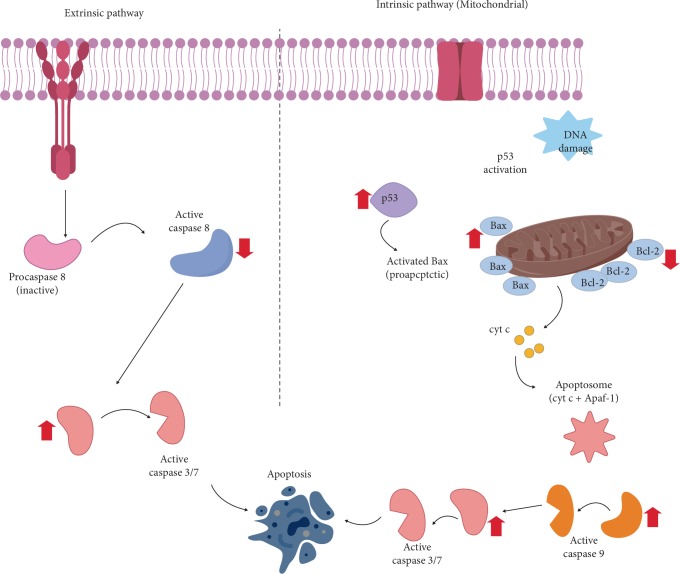
The proposed mechanism of apoptosis for the methanol extract of CA at 10 and 15 *µ*g/ml concentrations.

*Caffeic acid* has been reported to possess anticancer activity through its pro-oxidant property. Caffeic acid treatments have enhanced ROS levels and altered mitochondrial membrane potential in HeLa and ME-180 cancer cells [37]. *Rosmarinic acid* anticancer activity in many cancer cell has been reviewed by Swamy et al. [38]. *Eicosapentaenoid acid* induced apoptosis by activating caspase-3, inhibit cyclooxygenase-2 (COX-2) resulting in inhibition of prostaglandin synthesis and prostaglandin-mediated inflammatory process [39] Eiocosapentanoic acid (EFA) combined with other anticancer drugs caused a synergistic suppressor effect on TE-1 human esophageal cancer cell proliferations [40] (Figure 5).

*Benserazide*, a selective HK2 inhibitor was able to suppress cancer growth in tumor-bearing mice and inhibit glycolysis in aerobic glycolytic colorectal cancer cell SW 480 [41]. *Αlpha-linolenic acid* is an n-3-polyunsaturated fatty acid (PUFA) having an effective role in prostate cancer. Dietary of α- linolenic acid may trigger an increase in ALA, EPA, DPA, and DHA levels and significant decresase in arachidonic acid level during the mice's growth stage [42]. *Betaine* intake inversely associated with colorectal cancer risk [43].

*Salvianolic B* (Sal-B) significantly inhibited the growth of retinoblastoma cell HXO-RB44 and induced apoptosis with upregulation of caspase-3 expression and the induction of cell cycle arrest [44]. Sal-B was a leading bioactive compound in *Salvia Miltiorrhiza Bunge* and in vitro analysis showed that Sal-B could significantly reduce cell viability and suppress the proliferation of MDA-MB-231 and MCF-7 cells [45]. *4-Hydroxybenzoic acid* (4-HBA) inhibited cell proliferation of human K-562 leukemia cells and induced apoptosis with the inhibition of antiapoptotic activity of Bcl-2, Bcl-xl, and Mcl-1 [46]. 4 HBA also has anticancer activity in the MCF-7 breast cancer and induced apoptosis from the increased of expression of caspase-3 and PARP Cleavage, which was associated with the promotion of p 53 [47].

Treatment of *Firulic acid* isolated from *Ferula Foetida* decreased the viability, increased apoptosis, and suppressed the metastatic potential in breast cancer cell line MDA-MD-231 [48]. Ferulic acid significantly inhibited cell proliferation and induced arrest in G0/G1 phase of the cell cycle in Hela and Caski cells and also induced the cell cycle related protein expression of p53, P21 and reduced Cyclin D1 and cyclin E levels [49].

The phytochemical content of *C. amboinicus* or *P. amboinicus *has been characterized in many studies. Bhatt et al. [50], reported the major constituents of stem *P. amboinicus* in methanol extract were rosmarinic acid, caffeic acid, rutin, gallic acid, quercetin, and p-coumaric acid. Hemalatha et al. [51] who analyzed ethanolic extract of *P. amboinicus* leaves by GC-MS reported the major components were n-Hexadecanoic acid, thymol, 9-octadecenal (z), 10-heneicosane (c, t), and phytol. El-hawary et al. [52] also reported the major components in stems and roots of ethyl acetate extract of *P. amboinicus* grown in Egypt using UPLC-MS were caffeic acid, eriodyctiol, rosmarinic acid, coumaric acid, chrysoeriol and quercetin. Shubha and Bhatt [53] who analyzed the leaves using HPLC also reported that polyphenols and sugars were the dominant constituents and found chlorogenic acid, coumaric acid, and caffeic acid in appreciable concentrations. The solvent types, extraction technique, environmental condition of plant growth, and climate are several factors responsible for the variation of phytochemical content. From the result, we suggested that the presence of these bioactive compounds in CA would contribute to anticancer properties of this plant.

## 4. Conclusion

In conclusion, the present study demonstrates cytotoxicity of *C. amboinicus* to colon cancer. The extract showed potent anti-colon cancer activity against WiDr cells as compared to 5-flurouracil as a standard drug. The methanol extract of CA induces cell death at low concentrations mainly via apoptosis as upregulated several genes like BAX, P53 and also downregulated antiapoptotic gene like BCL2. A mode of cell death for the methanol extract of CA corresponded to apoptosis with intrinsic pathway in low concentrations as it upregulated *Caspase 9* but downregulated *Caspase 8*. On the other hand, at high concentrations might correspond to another programmed cell death. Therefore, further study related to other programmed cell death is important to validate the mechanism of action. The result obtained from LC MS analysis indicated the existences of many valuable bioactive compounds that would be contributed to anticancer properties of CA. *Coleus amboinicus* (Lour) shows potential as chemotherapeutic agents for colon cancer and might become an ingredient for health-beneficial foods to prevent colon cancer.

## Figures and Tables

**Table 1:** Primer sequences apoptosis related genes.

*Gene*	Forward and reverse primer
*BCL2 F * ^∗^	5′- GCT CTA AAA TCC ATC CAG -3′
*BCL2 R * ^∗^	5′- CCT CTC CAT CAT CAA CTT -3′
*BAX F * ^∗∗^	5′- CCC GAG AGG TCT TTT TCC GAG -3′
*BAX R * ^∗∗^	5′- CCA GCC CAT GAT GGT TCT GAT -3′
*Caspase 8 F * ^∗^	5′- AGA GTC TGT GCC CAA ATC AAC -3′
*Caspase 8 R * ^∗^	5′- GCT GCT TCT CTC TTT GCT GAA -3′
*Caspase 1 F * ^∗∗^	5′- CCT TCA AGG ACC TTG TCT GTT TAG -3′
*Caspase 1 R * ^∗∗^	5′- GAG GGA TTA CCC AAC TGT GAG -3′
*Caspase 9 F * ^∗∗^	5′- GAA TGA CGT GAA ACA CGA CAG -3′
*Caspase 9 R * ^∗∗^	5′- TTA ACG GCA TCC CCC ACT TAG -3′
*Caspase 7 F * ^∗∗^	5′- GCAGCGCCGAGACTTTTAG-3′
*Caspase 7 R * ^∗∗^	5′- GCTGCAGTTACCGTTCCCAC-3′
*TP53 F * ^∗∗^	5′ - CCG CAG TCA GAT CCT AGC -3′
*TP53 R * ^∗∗^	5′- AAT CAT CCA TTG CTT GGG ACG -3′
*ACTB F * ^∗∗^	5′- AGAGCTACGAGCTGCCTGAC -3′
*ACTB R * ^∗∗^	5′- AGCACTGTGTTGGCGTACAG -3′

References: ^∗^ [54]. ^∗∗^ [10].

**Table 2:** The expression of apoptosis-related genes after cells treated with 5-FU and methanol extract of CA at 10, 15, 25, and 50 *µ*g/ml concentrations.

Treatment	Fold change						
	Bax	Bcl-2	p53	Caspase 8	Caspase 9	Caspase 1	Caspase 7
Control	1.000 ± 0.000^a^	1.000 ± 0.000^a^	1.000 ± 0.000^a^	1.000 ± 0.000^a^	1.000 ± 0.000^a^	1.000 ± 0.000^a^	1.000 ± 0.000^a^
5-FU	1.800 ± 0.170^b^	0.236 ± 0.025^b^	3.285 ± 1.135^a^	0.325 ± 0.005^b^	4.785 ± 0.545^b^	0.445 ± 0.015^a^	0.235 ± 0.005^a^
10 *µ*g/ml	1.476 ± 0.138^b^	0.054 ± 0.010^b^	1.388 ± 0.402^a^	0.788 ± 0.095^b^	1.023 ± 0.412^a^	0.931 ± 0.084^a^	0.693 ± 0.082^a^
15 *µ*g/ml	1.665 ± 0.035^b^	0.807 ± 0.062^b^	1.887 ± 0.355^a^	0.710 ± 0.000^b^	1.263 ± 0.157^a^	1.130 ± 0.024^a^	1.020 ± 0.109^a^
25 *µ*g/ml	0.406 ± 0.023^b^	0.083 ± 0.013^b^	0.167 ± 0.001^a^	0.080 ± 0.029^b^	0.824 ± 0.119^a^	42.421 ± 2.057^b^	40.855 ± 7.147^b^
50 *µ*g/ml	0.773 ± 0.078^a^	0.043 ± 0.008^b^	0.050 ± 0.008^a^	0.046 ± 0.001^b^	0.825 ± 0.111^a^	34.253 ± 9.917^b^	37.888 ± 11.806^b^

^a^ *p* > 0.05 versus control.

^b^ *p* < 0.05 versus control.

**Table 3:** Phytochemicals of methanol extract of CA by Liquid Chromatography–Mass Spectrometry.

No	Name	Formula	RT [min]
1	Rosmarinic acid	C_18_ H_16_ O_8_	16.501
2	13,14-Dihydro-15-keto Prostaglandin J2	C_20_H_30_O_4_	23.906
3	7-Hydroxycoumarine	C_9_H_6_O_3_	19.616
4	L-(-)-Malic acid	C_4_H_6_O_5_	1.12
5	Pipecolic acid	C_6_H_11_N O_2_	0.886
6	cis-5,8,11,14,17-Eicosapentaenoic acid	C_20_H_30_O_2_	25.281
7	Ferulic acid	C_10_H_10_O_4_	15.391
8	Apigenin 7-O-glucuronide	C_21_H_18_O_11_	15.775
9	15-Deoxy-Δ12,14-prostaglandin A1	C_20_H_30_O_3_	25.554
10	13,14-Dihydro-15-keto Prostaglandin J2	C_20_H_30_O_4_	23.096
11	4-Hydroxybenzoic acid	C_7_H_6_O_3_	4.444
12	*α*-Lactose	C_12_H_22_O_11_	0.878
13	Apigenin 7-O-glucuronide	C_21_H_18_O_11_	15.974
14	Ferulic acid	C_10_H_10_O_4_	15.632
15	(2*α*,3*β*,19*α*)-2,3,19-Trihydroxyolean-12-en-28-oic acid	C_30_H_48_O_5_	22.265
16	1-Aminocyclohexanecarboxylic acid	C_7_H_13_N O_2_	0.882
17	Salvianolic acid B	C_36_H_30_O_16_	16.769
18	15-Deoxy-Δ12,14-prostaglandin A1	C_20_H_30_O_3_	24.459
19	Caffeic acid	C_9_H_8_O_4_	7.33
20	9S,13R-12-Oxophytodienoic acid	C_18_H_28_O_3_	23.569
21	Caffeic acid	C_9_H_8_O_4_	16.21
22	9-Oxo-10(E),12(E)-octadecadienoic acid	C_18_H_30_O_3_	23.983
23	Betaine	C_5_H_11_N O_2_	0.874
24	Caffeic acid	C_9_H_8_O_4_	16.491
25	*α*-Linolenic acid	C_18_H_30_O_2_	24.954

## Data Availability

The dataset used to support the funding of this study has been deposited in Mendeley Data (DOI: 10.17632/7trktkswrn.1).
